# Micra™ Leadless Intracardiac Pacemaker Implantation: A Safer Option During the Coronavirus Disease 2019 Pandemic

**DOI:** 10.19102/icrm.2021.120104

**Published:** 2021-01-15

**Authors:** Maryam Kazmi, Sana Rashid, Nebojsa Markovic, Hyoeun Kim, Emad F. Aziz

**Affiliations:** ^1^Department of Medicine, Rutgers New Jersey Medical School (NJMS), Newark, NJ, USA

**Keywords:** COVID-19, leadless pacing, Micra, pacemaker implantation

## Abstract

The Micra™ Transcatheter Pacing System (Medtronic, Minneapolis, MN, USA) is a fairly novel leadless intracardiac pacemaker implanted in the right ventricle via a femoral-vein transcatheter approach. Due to the less-invasive nature of the implantation procedure and its smaller size, patients receiving the Micra™ device tend to experience fewer complications, hospitalizations, and revisions when compared with those with transvenous pacemakers. Certain arrhythmias and conduction abnormalities, such as high-degree atrioventricular blocks, require urgent and timely pacemaker insertion—a necessity that has persisted even during the coronavirus disease 2019 (COVID-19) pandemic. Here, we present a case series of 10 patients with various conduction disease abnormalities who required right ventricle pacemaker implantation during the months of March to May 2020, which was the initial peak of the COVID-19 pandemic in New Jersey, including the enhanced precautions taken to avoid viral spread.

## Introduction

The Micra™ Transcatheter Pacing System (Medtronic, Minneapolis, MN, USA) is a fairly novel leadless intracardiac pacemaker that is implanted in the right ventricle via a femoral-vein transcatheter approach. Due to this device’s small size and the less-invasive nature of the implantation procedure, patients receiving the Micra™ device experience fewer complications, hospitalizations, and revisions as compared with those given transvenous pacemakers.^1^

Certain arrhythmias and conduction abnormalities, such as high-degree atrioventricular (AV) block, necessitate urgent and timely pacemaker insertion. This is true even in the context of the current coronavirus disease 2019 (COVID-19) pandemic, where enhanced precautions are mandated to limit viral spread. Here, we present a case series of 10 patients with various conduction disease abnormalities necessitating the implantation of a right ventricle pacemaker who presented during the months of March to May 2020, which was the time of the initial peak of the COVID-19 pandemic in New Jersey.

## Methods

Ten patients (six with AV block, two with symptomatic bradycardia, one with sinus node dysfunction, and one with symptomatic bradycardia and sinus node dysfunction) presented with various arrhythmias and conduction abnormalities during the COVID-19 pandemic to undergo pacemaker implantation at our institution with enhanced precaution protocols. The groin was prepped in the usual sterile manner. One percent lidocaine was used to infiltrate and anesthetize the groin area. According to our protocol from before the start of the pandemic, all Micra™ implantation patients would undergo light conscious sedation with midazolam (Versed^®^; Roche Holding, Basel, Switzerland). In this case series, no general anesthesia was used; instead, light conscious sedation was pursued in nine cases, while one patient was intubated and required no further sedation. The Micra™ introducer system was then inserted using the right or left femoral-vein approach after gradual upscale dilation and guidewire insertion at the entry site. The Micra™ delivery system was then advanced and deployed in the right ventricle “mid-septum” under fluoroscopy guidance. In this series, the Micra™ VR system (model MC1VR01) was used in two cases, while the Micra™ AV system (model MC1AVR1) was used in the remaining cases (n = 8). Fixation and electrical testing were performed to confirm the position. A standard sterile technique was maintained throughout the procedure. The patient’s face was masked and lightly covered with a sterile drape to reduce the spread of droplet and aerosol particles.

The operator remained at the femoral site, wearing proper personal protective equipment (PPE) and a sterile gown to minimize radiation as well as infectious exposure. The remainder of the electrophysiology staff also wore the correct PPE to reduce the chance of possible contagion spread.

## Results

Ten patients who had Micra™ leadless pacemakers implanted between March and May 2020 were included in this study **(Table 1)**. The majority of patients were male (n = 6) with an average age of 73.5 ± 12.2 years. Indications for the Micra™ pacemaker included high-degree AV block, sinus node dysfunction, and bradycardia with syncope. Of the 10 patients, all had hypertension, eight had hyperlipidemia, three had known paroxysmal atrial fibrillation, and one had heart failure. Three of the patients tested positive for COVID-19 and were noted to be hypoxic during hospitalization, while five of the patients tested negative and two were not tested. Of note, the two patients who were not tested for COVID-19 had the earliest procedure dates; testing was performed on all patients operated on after the first week of April 2020. The average total procedure time was 30.3 ± 11.8 minutes and the average fluoroscopy time was 2.6 ± 0.8 minutes. The average total dose-area product during the procedures was 914.4 ± 992.4 Gy·cm.^2^ Postprocedure, one patient developed a femoral artery pseudoaneurysm, which was managed with a thrombin injection. One COVID-19 patient experienced in-hospital mortality on the third postoperative day secondary to hypoxic respiratory failure triggered by COVID-19.

## Discussion

Enhanced procedure precautions are paramount during the ongoing COVID-19 pandemic considering the high transmissibility of the severe acute respiratory syndrome coronavirus 2 (SARS-CoV-2) virus. At the same time, pacemakers are life-saving devices and implantation should not be delayed in patients with conduction abnormalities.

Here, we propose the use of leadless pacemakers—in this case, the Micra™ system—to reduce the chance of exposure to contagion among the operator, staff, and patient. This was possible due to several factors. First, the Micra™ device is inserted via the femoral vein, allowing for increased distance to be maintained from the patient’s respiratory droplets and aerosols. Second, implantation of the Micra™ system is itself a less-invasive procedure, allowing for shorter time to be spent in the electrophysiology lab and a reduced mean fluoroscopy time (2.7 minutes) as compared with a mean fluoroscopy time for single-chamber transvenous pacers of 5.5 minutes.^2^ Moreover, due to its smaller size and less-invasive approach, implantation of the Micra™ system was associated with fewer hospitalizations; system revisions; and complications such as pneumothorax, device emboli, dislodgement, and infections relative to traditional transvenous pacemakers at six months of follow-up, with comparable levels of efficacy observed for both.^1^

Even though only three patients tested positive for COVID-19 in this series, enhanced droplet and aerosol precautions were maintained for all patients given the unknown false-negative rate of the real-time polymerase chain reaction SARS-CoV-2 RNA test. Two of our patients were not tested due to a lack of readily available testing during the early months of the pandemic. Until more sensitive testing is developed, enhanced precautions must be maintained for all patients.

One limitation to the widespread application of the Micra™ device is that it is a ventricular-only pacing device and thus only can serve a limited number of patients with conduction abnormalities.^1^ However, when deemed appropriate, the Micra™ device should be favored to reduce the transmission of SARS-CoV-2 while also decreasing radiation exposure and the rate of complications.

## Figures and Tables

**Table 1:** Characteristics of Patients Who Underwent Micra™ Device Implantation

Pt.	Age (Years)	Sex	HTN	HLD	HF	SARS-CoV-2 Status	PPM Indication	Micra™ Model
1	66	M	Y	Y	N	(+)	AV block	AV
2	69	M	Y	N	Y	(−)	AV block	VR
3	87	F	Y	Y	N	Not tested	AV block	AV
4	88	F	Y	Y	N	(−)	Symptomatic bradycardia	AV
5	51	M	Y	Y	N	(−)	Sinus node dysfunction	VR
6	71	M	Y	Y	N	Not tested	AV block	AV
7	65	M	Y	N	N	(+)	AV block	AV
8	85	F	Y	Y	N	(−)	Sinus node dysfunction, symptomatic bradycardia	AV
9	88	F	Y	Y	N	(−)	Symptomatic bradycardia	AV
10	65	M	Y	N	N	(+)	AV block	AV

AV: atrioventricular; HF: heart failure; HLD: hyperlipidemia; HTN: hypertension; PPM: permanent pacemaker; SARS-CoV-2: severe acute respiratory syndrome coronavirus 2.

## References

[1] Reynolds DW, Ritter P (2016). A leadless intracardiac transcatheter pacing system.. N Engl J Med.

[2] Larsen TR, Saini A, Moore J (2019). Fluoroscopy reduction during device implantation by using three-dimensional navigation. A single-center experience.. J Cardiovasc Electrophysiol.

